# Role of glucose and ketone bodies in the metabolic control of experimental brain cancer

**DOI:** 10.1038/sj.bjc.6601269

**Published:** 2003-09-30

**Authors:** T N Seyfried, T M Sanderson, M M El-Abbadi, R McGowan, P Mukherjee

**Affiliations:** 1 Biology Department, Boston College, Chestnut Hill, MA 02467, USA

**Keywords:** ketogenic diet, caloric restriction, IGF-1, glioma, angiogenesis, metabolic control

## Abstract

Brain tumours lack metabolic versatility and are dependent largely on glucose for energy. This contrasts with normal brain tissue that can derive energy from both glucose and ketone bodies. We examined for the first time the potential efficacy of dietary therapies that reduce plasma glucose and elevate ketone bodies in the CT-2A syngeneic malignant mouse astrocytoma. C57BL/6J mice were fed either a standard diet unrestricted (SD-UR), a ketogenic diet unrestricted (KD-UR), the SD restricted to 40% (SD-R), or the KD restricted to 40% of the control standard diet (KD-R). Body weights, tumour weights, plasma glucose, *β*-hydroxybutyrate (*β*-OHB), and insulin-like growth factor 1 (IGF-1) were measured 13 days after tumour implantation. CT-2A growth was rapid in both the SD-UR and KD-UR groups, but was significantly reduced in both the SD-R and KD-R groups by about 80%. The results indicate that plasma glucose predicts CT-2A growth and that growth is dependent more on the amount than on the origin of dietary calories. Also, restriction of either diet significantly reduced the plasma levels of IGF-1, a biomarker for angiogenesis and tumour progression. Owing to a dependence on plasma glucose, IGF-1 was also predictive of CT-2A growth. Ketone bodies are proposed to reduce stromal inflammatory activities, while providing normal brain cells with a nonglycolytic high-energy substrate. Our results in a mouse astrocytoma suggest that malignant brain tumours are potentially manageable with dietary therapies that reduce glucose and elevate ketone bodies.

The long-term prognosis remains poor for most patients with malignant brain tumours despite advances in the molecular genetics of cancer and in brain imaging techniques (Shapiro, 1999). Surgical resection followed by radiation is the standard therapy today as it has been for over five decades. Chemotherapy also has had little positive benefit on malignant glioma management and is often associated with adverse effects that diminish quality of life (Shapiro, 1999). Although therapeutic targeting of tumour-associated mutations may be effective in tumour management, most tumour mutations arise as later stage epiphenomena of tissue disorganisation and their involvement with tumour initiation, promotion, or progression has not been conclusively established (Sonnenschein and Soto, 1999,^2000^; Seyfried, 2001; Bergstein, 2003). Clearly, alternative therapies are needed that can better manage brain tumours while permitting a decent quality of life.

Metabolic control theory applies principles of bioenergetics for the control or management of complex diseases (Veech *et al*, 2001; Strohman, 2002; Greene *et al*, 2003). Since metabolism is a universal process underlying all phenotypes, modification of metabolism can potentially modify phenotype. The theory is based on the idea that compensatory genetic and biochemical pathways regulate the bioenergetic potential of glycolysis, the tricarboxylic acid (TCA) cycle, and the electron transport chain. This produces a flexible and versatile metabolic system that is capable of restoring an orderly adaptive behaviour to widely disordered conditions involving complex gene–environmental interactions (Greenspan, 2001; Strohman, 2002; Greene *et al*, 2003). As biological chaos underlies the progression of brain tumours (Seyfried, 2001), principles of metabolic control theory may be effective in managing brain cancer.

In 1995, Nebeling *et al* reported that a ketogenic diet could manage advanced stage malignant astrocytoma in two female paediatric patients (Nebeling *et al*, 1995). The ketogenic diet (KD) is a high-fat, low-protein, low-carbohydrate diet that has been used for decades to treat patients with refractory epilepsies (Freeman *et al*, 2000; Todorova *et al*, 2000; Greene *et al*, 2003). It was not clear, however, whether KD controlled paediatric astrocytoma through effects on plasma glucose or ketone bodies since the diet was administered under restricted conditions where blood glucose levels were also reduced (Nebeling *et al*, 1995). Although the findings with paediatric astrocytoma generated considerable interest in the brain tumour field (Nebeling and Lerner, 1995), no further studies were conducted in humans and no studies have evaluated the efficacy of this dietary therapy in a brain tumour animal model.

While brain cells metabolise glucose for energy under normal physiological conditions, they can metabolise ketone bodies (acetoacetate and *β*−hydroxybutyrate) for energy when blood glucose levels decrease as occurs during fasting or caloric restriction (Owen *et al*, 1967; Clarke and Sokoloff, 1999; Greene *et al*, 2001,^2003^). In contrast to glucose, ketone bodies bypass cytoplasmic glycolysis and directly enter the TCA cycle as acetyl CoA (Sato *et al*, 1995; Veech *et al*, 2001). Gliomas and most tumour cells, however, lack this metabolic versatility and are largely dependent on glycolytic energy (Fearon *et al*, 1988; Mies *et al*, 1990; Oudard *et al*, 1997; Aronen *et al*, 2000; Roslin *et al*, 2003). Defects in ketone body metabolism, the mitochondrial TCA cycle, and electron transport chain systems are thought to underlie the dependence of tumour cells on glycolytic energy (Warberg, 1956; Fredericks and Ramsey, 1978; Tisdale and Brennan, 1983; Lichtor and Dohrmann, 1986; John, 2001). Hence, therapies that exploit the genetic and metabolic weakness of brain tumour cells may be effective in controlling brain cancer.

Dietary caloric restriction (DR) has long been recognised as a natural therapy that improves health, promotes longevity, and significantly reduces the incidence, establishment, and growth of many tumour types (Rous, 1914; Tannenbaum, 1959; Weindruch and Walford, 1988; Birt *et al*, 1999; Kritchevsky, 1999a; Duan *et al*, 2003; Koubova and Guarente, 2003). Dietary caloric restriction is produced from a total dietary restriction and differs from acute fasting or starvation in that DR reduces total caloric energy intake without causing anorexia or malnutrition (Tannenbaum, 1959; Mukherjee *et al*, 1999). We recently showed that a moderate DR of 30–40% significantly reduced angiogenesis and growth in the CT-2A malignant mouse astrocytoma model (Mukherjee *et al*, 2002). Moreover, DR significantly increased the number of apoptotic cells in the CT-2A tumour. We suggested that DR reduced CT-2A growth through effects on both the tumour cells and on the tumour-associated host cells. Reduced glycolytic energy together with a global downregulation of inflammatory and angiogenic properties of the microenvironment were proposed to underlie the antitumour effects of DR (Mukherjee *et al*, 1999,^2002^).

In this study, we compared the effects of restricted feeding of a standard diet or a KD on the orthotopic growth of the CT-2A brain tumour. While CT-2A growth was rapid with the unrestricted feeding of either diet, a moderate 40% restriction of either diet significantly reduced growth. Reductions of plasma glucose together with elevations of plasma ketones were associated with the antitumour efficacy of the diets. Also, restriction of either diet significantly reduced the plasma levels of insulin-like growth factor-1 (IGF-1), a biomarker for angiogenesis and tumour progression. Our results in mice suggest that malignant brain tumours may be manageable in part through dietary reduction of glucose and elevation of ketone bodies.

## MATERIALS AND METHODS

### Mice

Mice of the C57BL/6J strain were obtained from Jackson Laboratory (Bar Harbor, ME, USA). The mice were propagated in the animal care facility of Department of Biology of Boston College. Male mice (10–12 weeks of age) were used for the studies and were provided with food under either *ad libitum* or unrestricted (UR) conditions or under restricted (R) conditions (as below). Water was provided *ad libitum* to all mice. The animal room was maintained at 22±1°C and cotton nesting pads were provided for additional warmth. All animal experiments were carried out with ethical committee approval in accordance with the National Institutes of Health Guide for the Care and Use of Laboratory Animals and were approved by the Institutional Animal Care Committee. These procedures also met the standards required by the UKCCCR guidelines (Workman *et al*, 1998).

### Brain tumour model

The syngeneic CT-2A experimental mouse brain tumour used for these studies was the second cerebral tumour (CT-2) generated in our laboratory after implantation of 20-methylcholanthrene into the cerebral cortex of a C57BL/6J mouse according to the procedure of Zimmerman (Zimmerman and Arnold, 1941; Seyfried *et al*, 1992). Histologically, the CT-2A brain tumour is broadly classified as a poorly differentiated highly malignant anaplastic astrocytoma (Seyfried *et al*, 1992). The tumour grows orthotopically as a soft, noncohesive, and highly vascularised mass.

### Intracerebral tumour implantation

The CT-2A tumour was implanted into the cerebral cortex of C57BL/6J mice using a trocar as we previously described (Seyfried *et al*, 1987; Ranes *et al*, 2001). Briefly, mice were anaesthetised with 2, 2, 2-tribromoethanol intraperitoneally and their heads were shaved and swabbed with 70% ethyl alcohol under sterile conditions. Small CT-2A tumour pieces from a C57BL/6J donor mouse were matched for size (about 1 mm^3^) based on a grid and were implanted into the right cerebral hemisphere of anaesthetised recipient mice (Ranes *et al*, 2001). Initiation of tumours from intact tumour pieces is preferable to initiation from cultured cells since the pieces already contain an established microenvironment and vasculature that facilitates rapid tumour establishment and growth (Tannenbaum, 1959). All of the mice recovered completely from the surgical procedure and were returned to their cages when fully active (within 2 h). No animals died from either the surgical procedures or from tumour growth. All mice were euthanised prior to tumour morbidity.

### Diets

All mice received PROLAB chow (Agway Inc.,. NY, USA) prior to the experiment. This is the standard diet (SD) and contained a balance of mouse nutritional ingredients. According to the manufacturer's specification, this diet delivers 4.4 kcal g^−1^ gross energy, where fat, carbohydrate, protein, and fibre comprised 55, 520, 225, and 45 g kg^−1^ of the diet, respectively. The ketogenic diet was obtained from Zeigler Bros., Inc. (Gardners, PA, USA) and also contained a balance of mouse nutritional ingredients. According to the manufacturer's specification, the KD delivers 7.8 kcal g^−1^ gross energy, where fat, carbohydrate, protein, and fibre comprised 700, 0, 128, and 109 g kg^−1^ of the diet, respectively. The fat in this diet was derived from lard and the diet had a ketogenic ratio (fats : proteins+carbohydrates) of 5.48 : 1.

### Dietary restriction

The mice were group housed prior to the initiation of the experiment and were then housed singly in plastic shoebox cages 1 day before tumour implantation. After tumour implantation, mice were randomly assigned to one of four diet groups that received either: (1) the standard diet fed *ad libitum* or unrestricted (SD-UR), (2) the KD fed *ad libitum* or unrestricted (KD-UR), (3) the SD restricted to 40% (SD-R), or (4) the KD restricted to 40% of the control standard diet (KD-R). Total dietary restriction maintains a constant ratio of nutrients to energy, that is, the average daily food intake (g) for the UR-fed mice was determined every other day and the R-fed mice were given 60% of the SD-UR group on a daily basis. This ensured that the mice in both R mouse groups received a similar number of total calories throughout the study. The dietary treatments were initiated 24 h following tumour implantation and were continued for 13 days. Body weights of all mice were recorded every other day.

### Tumour growth

Intracerebral tumour growth was analysed directly by measuring total tumour dry weight. Tumours were dissected from normal appearing brain tissue, frozen, and then lyophilised to remove water. From our experience, total tumour dry weight is a more accurate measure of tumour growth than total wet weight because individual CT-2A tumours can vary in the degree of haemorrhage and oedema.

### Measurement of plasma glucose and *β*-hydroxybutyrate

Blood was collected from mice on the last day of the experiment and before tumour resection. The mice were anaesthetised with Isoflurane (Halocarbon), and blood was collected from the heart in heparinised tubes. All mice were fasted for 3 h before blood collection. The blood was centrifuged at 1600 **g** for 10 min, and the plasma was collected and stored at −80°C until assayed. Plasma glucose and *β-*hydroxybutyrate (*β*-OHB) concentrations were measured spectrophotometrically using the Trinder assay and a UV enzymatic assay, respectively (Sigma, St Louis, MO, USA). We measured only *β*-OHB levels because *β*-OHB is derived from acetoacetate in the liver and is the major blood ketone body (Krebs *et al*, 1971; Bhagavan, 2002).

### Insulin-like growth factor-1 (IGF-1) analysis

Plasma IGF-1 concentrations were measured by radioimmunoassay (Nichols Institute Diagnostics, Capistrano, CA, USA) with purified IGF-1 as the standard and controls supplied by kit as we previously described (Mukherjee *et al*, 1999).

### Statistical analysis

Body weight, food intake, tumour growth and all serum quantitative measurements were analysed by ANOVA followed by Fisher's PLSD to calculate two-sided pairwise comparison among different test groups by use of Statview 5.0. In each figure, *n* designates the number of individual mice analysed. Error bars in the figures are expressed as 95% confidence intervals (CIs) (1.96 × standard error of mean) according to the recommended standards (Streiner, 1996). Linear regression was used to determine the relationships among plasma glucose, *β*-OHB, and tumour growth (Lang and Secic, 1997). The regressions are expressed as the slope±95% CI.

## RESULTS

No adverse effects were observed in the 40% R-fed mice that were maintained on either the SD or the KD. Although total body weight was reduced, the R-fed mice were healthy and more physically active than the mice in either UR-fed group. No signs of vitamin or mineral deficiency were observed in the R-fed mice according to standard criteria for mice (Hoag and Dickie, 1968). These findings are consistent with the well-recognised health benefits of mild-to-moderate diet restriction in rodents (Tannenbaum, 1959; Weindruch and Walford, 1988; Keenan *et al*, 1999). The UR-KD mice also appeared healthy throughout the study, but displayed oily fur and stool discoloration (yellowish). The intestinal tract was also discolored in UR-KD mice upon dissection.

### Energy intake

**Figure 1:**
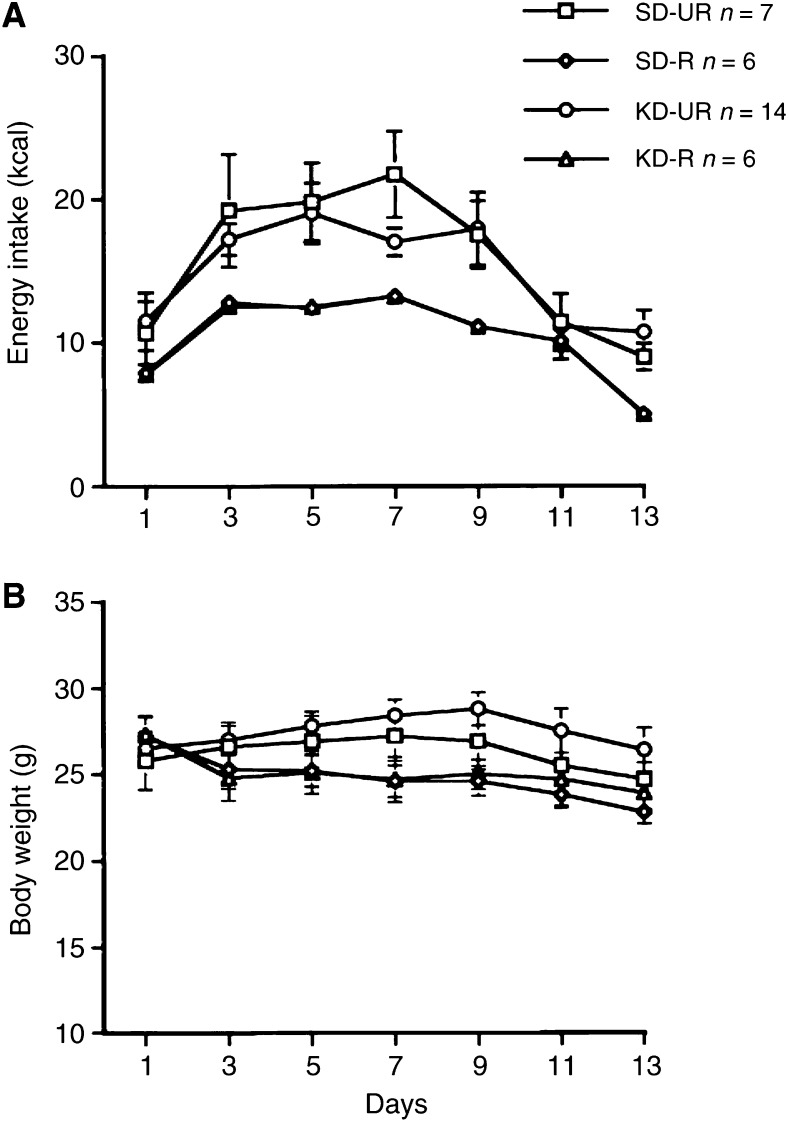
Energy intake (**A**) and body weights (**B**) in male C57BL/6J mice bearing the intracerebral CT-2A brain tumour. Tumours were implanted on day 0 and dietary treatment was started on day 1. Values are expressed as means with 95% confidence intervals, and *n*=the number of tumour bearing mice examined in each group.

Energy intake increased significantly about 2 days after intracerebral tumour implantation in both UR-fed groups (Figure 1A

). This phenomenon was also observed previously in mice pretreated with DR for 7 days prior to tumour implantation (Mukherjee *et al*, 2002). This increased energy intake results from hyperphagia (binge eating) that may be associated with cerebral hyperglycolysis following tumour implantation (Gadisseux *et al*, 1984; Bergsneider *et al*, 1997; Mukherjee *et al*, 2002). In order to maintain a 40% dietary restriction throughout the study, energy intake was increased in the R-fed mice to compensate for the hyperphagia in the UR-fed mice.

The initial total energy intake of the UR-fed groups was about 11–12 kcal day^−1^, but rose to about 20 kcal day^−1^ during the hyperphagic period (Figure 1A). Energy intake declined in these mice after about day 9 and returned to the initial intake by day 11. The reduced energy intake beyond day 11 reflected the effects of tumour burden. The total energy intake of the R-fed groups was adjusted to 60% of that in the UR-SD group throughout the study. Thus, both R-fed groups received similar amounts of total calories despite differences in the caloric origin of the diets.

The hyperphagia in the UR-fed groups was not associated with a significant increase in body weight (Figure 1B). A similar finding was seen in sham-operated mice suggesting that the brain trauma from tumour implantation increased basal metabolic rate. The R-fed mice lost about 10% of their initial body weight during the first week of treatment and their weights remained significantly lower than those of the UR-fed groups throughout the study (*P*<0.001). The body weight decline in the R-fed groups after day 9 reflected the body weight decline in the UR-fed groups due to increased tumour burden.

### Dietary restrictions reduce intracerebral CT-2A tumour growth

**Figure 2:**
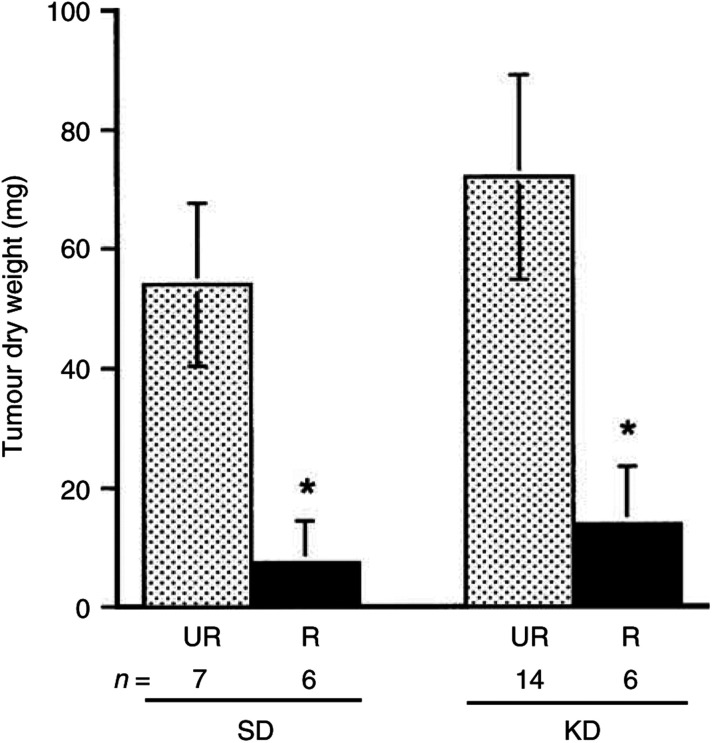
Influence of diet on the intracerebral growth of the CT-2A brain tumour. Dietary treatment was initiated 1 day after tumour implantation and was continued for 13 days as shown in Figure 1. Tumour weights were measured in C57BL/6J mice receiving either the standard diet (SD) or ketogenic diet (KD) under either unrestricted (UR) or restricted (R) feeding. Values are expressed as means with 95% CIs, and *n*=the number of mice examined in each group. The dry weights of the tumours in R groups were significantly lower than those in the UR groups at *P*<0.01.

The intracerebral CT-2A tumour grew rapidly and to a large size in both groups of UR-fed mice (Figure 2

). Restricted feeding of either the SD or the KD significantly reduced tumour growth. The mean tumour dry weight was approximately 86**%** less in the SD-R group than in the SD-UR group and was approximately 80**%** less in the KD-R group than in the KD-UR group (Figure 2). This growth reduction in tumour weight greatly exceeded the reduction in the body weight. It is important to mention that all implanted tumours grew in both the UR- and R-fed groups suggesting that restricted feeding of either the SD or the KD did not prevent tumour ‘take’ or establishment, but significantly reduced the intracerebral growth of the malignant CT-2A brain tumour.

### Influence of diet on plasma glucose and *β*-OHB levels

Plasma glucose levels were significantly lower in the R-fed mouse groups than in the UR-fed groups (Table 1

**Table 1:** Influence of diet on plasma glucose, *β*-OHB, and IGF-1 levels in mice bearing the CT-2A intracerebral brain tumour

**Diet**^b^	**Groups**^c^	**Glucose (mmol l^−1^)**	***β*-OHB (mmol l^−1^)**	**IGF-1 (ng ml^−1^)**
SD	UR	9.1±0.9	0.6±0.1	208±25
		(7)^d^	(7)	(6)
	R	5.2±1.1^*^	1.4±0.2^*^	117±36^*^
		(6)	(6)	(6)
KD	UR	11.4±1.4	1.0±0.3	294±30
		(14)	(14)	(5)
	R	5.7±1.5^*^	1.3±0.6	193±57^*^
		(6)	(6)	(6)

a Values are expressed as means ±95% confidence intervals as described in Materials and Methods.

b Animals were fed either a standard chow diet (SD) or a ketogenic diet (KD).

c UR(unrestricted feeding) and R (restricted to 60% of the SD-UR group as described in Materials and Methods).

d Numbers in parentheses indicate the number of independent tumour-bearing mice examined in each group. The asterisks indicate that the values of the R groups differed from those of their respective UR groups at *P* < 0.01 (analysed by ANOVA, one-way).

). Plasma glucose levels, however, were similar in both UR-fed mouse groups. These findings are consistent with previous studies in mice that the KD, fed *ad libitum*, does not significantly alter plasma glucose levels (Todorova *et al*, 2000). In contrast to glucose levels, *β*-OHB levels were two-fold greater in the SD-R mice than in the SD-UR mice (Table 1). Although the ketone levels in the KD-UR and KD-R groups were not significantly different, the ketone levels in both these groups were significantly greater than that in the SD-UR. These findings are consistent with previous studies in mice that *β*-OHB levels are increased under caloric restriction or the KD (Todorova *et al*, 2000; Greene *et al*, 2001).

### Statistical analysis of plasma glucose levels, *β*-OHB levels, and CT-2A tumour growth

To determine whether blood glucose levels were predictive of blood *β*-OHB levels and tumour growth, we analysed the data using simple linear regression. These statistical analyses also included one mouse considered an outlier, that is, a mouse that ate less in the SD-UR group. This mouse also had reduced body weight and plasma glucose. This outlier was considered restricted for caloric intake and similar to the mice in R-fed groups. Although the outlier was excluded from the data presented in Figures 1 and 2 and Table 1, the data from this mouse were included in the regression analyses in order to assess the relationships between glucose, *β*-OHB, and tumour growth.

Simple linear regression analysis was used to examine the relationship between plasma glucose levels (the independent or explanatory *X* variable) and *β*-OHB levels (the dependent or response *Y* variable) for both the SD and the KD. These variables were identified based on physiological and neurochemical studies showing that plasma glucose levels determine plasma *β*-OHB levels during periods of fasting (Clarke and Sokoloff, 1999; Greene *et al*, 2001). The assumptions of simple linear regression were met according to the established criteria (Lang and Secic, 1997). The slopes of the regression lines were highly significant (*P*<0.01) and showed that plasma *β*-OHB levels increased as glucose levels decreased for the SD (−0.143±0.074; *t*=−4.22; *Y*=2.03–0.143*X*, *n*=14) and for the KD (−0.099±0.063; *t*=−3.26; *Y*=2.028–0.099*X*, *n*=20). The coefficient of determination (*r*^2^) was greater in the SD group (0.598) than in the KD group (0.384). This is expected since ketone levels are higher in the KD-UR mice than in the SD-UR mice and this would reduce the association. Viewed together, these findings indicate that plasma *β*-OHB levels are dependent on plasma glucose levels and that glucose levels predict *β*-OHB levels in both dietary groups.

**Figure 3:**
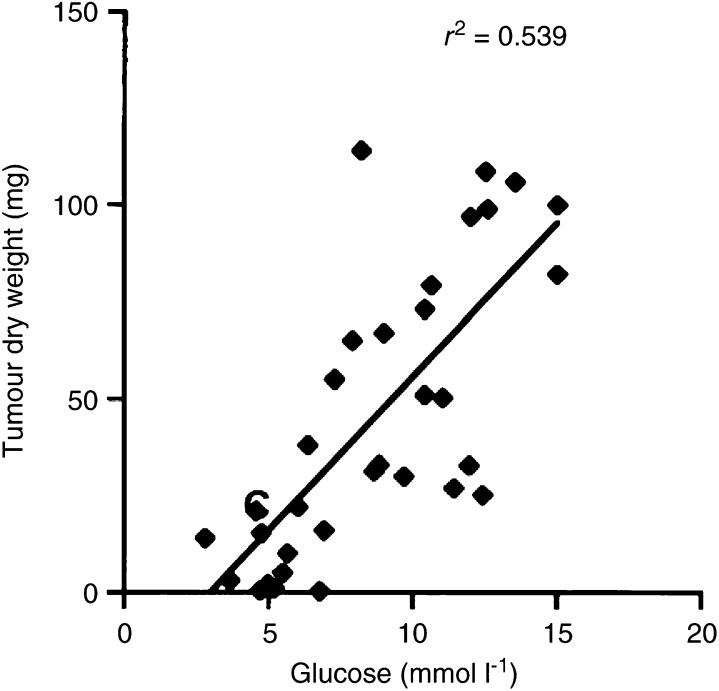
Linear regression analysis of plasma glucose and CT-2A-tumour growth in mice from both the SD and KD dietary groups combined (*n*=34). These analyses included the values for plasma glucose and tumour growth of individual mice from both the UR- and R-fed groups. The linear regression was highly significant at ^*^P<0.001.

We next used linear regression to analyse the association between plasma glucose (the *X* variable) and CT-2A growth (the *Y* variable) for both dietary groups combined (Figure 3

). The slope of the regression line was highly significant (7.96+2.75; *t*=5.90; *Y*=−23.84±7.96X, *n*=34) (Figure 3). Thus, plasma glucose levels were highly significant in predicting CT-2A growth.

Since the above linear regression indicated that *β*-OHB levels were dependent on glucose levels, the slope was significant for the association between CT-2A growth and *β*-OHB levels in the SD group (*r*^2^=0.637), but not for the KD group (*r*^2^=0.085). Hence, no association was seen between CT-2A growth and blood ketone levels (Figure 2 and Table 1) indicating that ketone elevation alone is unable to reduce growth.

### Plasma IGF-1

**Figure 4:**
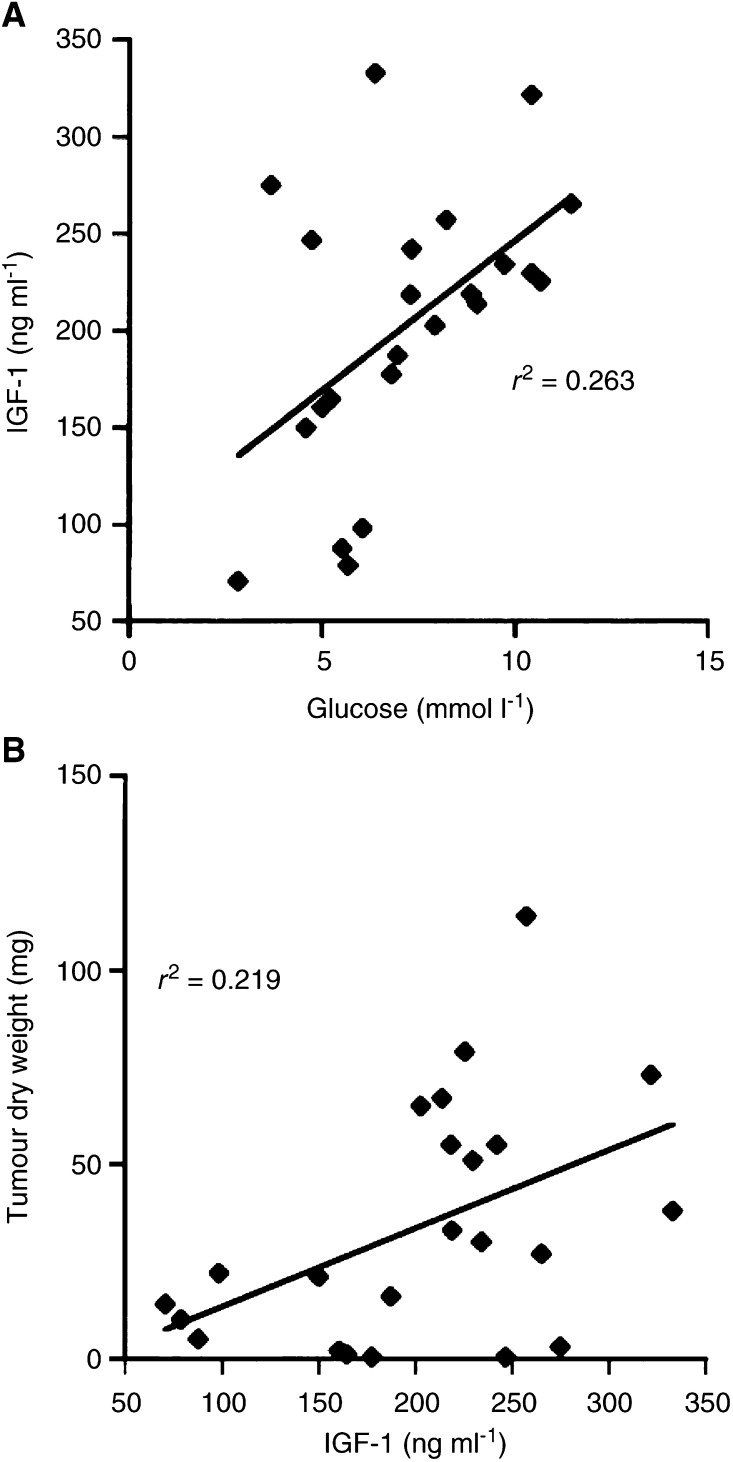
Linear regression analysis of plasma glucose and IGF-1 levels (**A**), and plasma IGF-1 levels and CT-2A-tumour growth (**B**) in mice fed with both the SD and KD (*n*=23). These analyses included the values for plasma glucose and *β*-OHB levels of individual mice from both the UR- and R-fed groups. The linear regressions were highly significant at *P*<0.01.

Circulating IGF-1 levels were significantly lower in each R-fed mouse group than in the respective UR-fed group (Table 1). Changes in plasma IGF-1 levels were also associated with changes in plasma glucose levels (Table 1). Indeed, linear regression analysis indicated that plasma glucose is predictive of plasma IGF-1 levels (Figure 4A

). The slope of the regression line was significant and showed that IGF-1 decreased as glucose levels decreased (15.42±11.68; *t*=2.74; *Y*=92.20+15.42*X*). Since plasma glucose levels are predictive of both tumour growth and IGF-1 levels, we next analysed the relationship between plasma IGF-1 levels and CT-2A tumour growth (Figure 4B). The slope of this regression line was also significant and showed that CT-2A tumour growth decreased as IGF-1 levels decreased (0.20±0.02; *t*=2.43; *Y*=−6.84+0.20*X*). These findings indicate that plasma IGF-1, like plasma glucose, is predictive of CT-2A growth.

## DISCUSSION

Normal mammalian brain cells are metabolically versatile and capable of deriving energy from glucose and ketone bodies (Greene *et al*, 2003). Although the levels of glucose and ketones in brain are proportional to their levels in blood, the adult brain does not usually metabolise ketones for energy under normal physiological conditions (Clarke and Sokoloff, 1999; Laterra *et al*, 1999; Guzman and Blazquez, 2001; Bhagavan, 2002). The brain will, however, actively metabolise ketone bodies for energy if blood glucose levels are reduced. We recently showed that circulating ketone levels are inversely related to circulating glucose levels in calorie restricted epileptic mice (Greene *et al*, 2001). The results of the present study support these previous findings. Ketone bodies bypass cytoplasmic glycolysis and directly enter the TCA cycle as acetyl CoA (Nehlig and Pereira de Vasconcelos, 1993; Sato *et al*, 1995; Kashiwaya *et al*, 2000; Veech *et al*, 2001). This causes significant increases in the TCA cycle metabolites and improves physiological performance through an increase in the energy of ATP hydrolysis (Sato *et al*, 1995; Veech *et al*, 2001).

We suggest that the metabolic mechanism by which moderate DR inhibits CT-2A brain tumour growth involves a failure to utilise alternative energy substrates. Most tumours including primary brain tumours actively consume glucose and are largely dependent on glycolysis for energy (Warberg, 1956; Galarraga *et al*, 1986; Mies *et al*, 1990; Oudard *et al*, 1997; Roslin *et al*, 2003). Mitochondrial defects and an inability to metabolise ketone bodies are thought to be responsible for the dependence of tumour cells on glycolytic energy (Warberg, 1956; Fredericks and Ramsey, 1978; Pedersen, 1978; Tisdale and Brennan, 1983; Lichtor and Dohrmann, 1986; Oudard *et al*, 1997; John, 2001). The flexibility and versatility of gene-linked metabolic network interactions in response to alterations in nutritional environment underlies metabolic control theory (Greenspan, 2001; Veech *et al*, 2001; Strohman, 2002; Greene *et al*, 2003). As DR inhibits glycolysis, DR would force cells to switch from glucose to alternative noncarbohydrate energy metabolites, for example, ketones (Greene *et al*, 2003; Hagopian *et al*, 2003). While this switch occurs readily in normal cells, the switch is more difficult for tumour cells due to their accumulated genetic defects. Our results in the CT-2A astrocytoma support the feasibility of metabolic control for the management of brain cancer.

CT-2A growth reduction was associated with reduced blood glucose levels. Indeed, linear regression analysis showed that blood glucose levels were predictive of CT-2A growth. Although blood ketone levels were elevated under restriction of either diet, elevated ketone levels alone could not account for reduced tumour growth because tumour growth was rapid in the UR-KD group despite the presence of high ketone levels. These findings support the previous observations of Fearon *et al* (1985), who showed that the failure of a KD to restrict growth of the Walker 256 rat tumour resulted from the failure of ketosis to reduce glucose availability. Moreover, reduced blood glucose was associated with the management of advanced stage malignant astrocytoma in two female paediatric patients with the KD (Nebeling and Lerner, 1995; Nebeling *et al*, 1995). Hence, reduced glucose is a key factor in the metabolic control of the mouse CT-2A tumour and possibly human paediatric astrocytoma.

If the antitumour effects of restricted caloric intake are associated with reduced glucose levels and glycolytic energy, a question arises as to what role elevated ketone levels might have in CT-2A management. We suggest that ketone body metabolism, while providing normal brain cells with an alternative high-energy substrate, also reduces the inflammatory activities of tumour-associated host cells (stromal cells). Ketone body metabolism reduces oxygen free radicals, enhances tolerance to hypoxia, and may prevent organ dysfunction from inflammatory processes (Kirsch and D'Alecy, 1984; Yu *et al*, 1990; Marcondes *et al*, 2001; Veech *et al*, 2001; Sharman *et al*, 2002). Indeed, Dong *et al* (1998) reported that moderate caloric restriction could reduce the proinflammatory properties of macrophages while enhancing their phagocytic function. Activated macrophages also contribute to tumour angiogenesis that is reduced under DR (Mukherjee *et al*, 1999,^2002^). Uncoupling the detrimental inflammatory activities of macrophages from their potentially beneficial phagocytic activities is considered important for the eventual management of brain cancer (Seyfried, 2001). Hence, a shift in energy metabolism from glucose to ketones will enhance the bioenergetic potential of normal brain cells on the one hand while reducing tumour cell growth and tumour inflammatory properties on the other hand.

Insulin-like growth facor-1 is considered a biomarker for angiogenesis and tumour progression (Kari *et al*, 1999; Mukherjee *et al*, 1999; Yu and Rohan, 2000). Besides predicting CT-2A growth, we found that plasma glucose also predicts plasma IGF-1 levels. These observations agree with previous studies that glucose regulates IGF-1 expression (Straus and Burke, 1995; Wang *et al*, 2000). We recently presented histological evidence that moderate dietary restriction significantly reduces vascularity (angiogenesis) in the CT-2A brain tumour (Mukherjee *et al*, 2002). Our findings that moderate restriction of either the SD or the KD reduced circulating IGF-1 levels support further the antiangiogenic effects of DR. Since plasma IGF-1 and glucose are both predictive of CT-2A tumour growth, we suggest that either glucose or IGF-1 may be useful biomarkers for predicting the effects of DR on brain tumour growth and angiogenesis.

In addition to reducing angiogenesis, moderate DR also enhances apoptosis in the CT-2A tumour and in prostate cancer (Mukherjee *et al*, 1999,^2002^). This is the interesting in light of recent findings that the glycolysis inhibitor 2-deoxy-D-glucose or glucose deprivation enhances apoptosis in human breast and lung cancer cells (Lee *et al*, 1997; Shim *et al*, 1998; Aft *et al*, 2002). Since DR also reduces blood glucose levels, it is possible that DR reduces CT-2A tumour growth through glucose-dependent apoptotic pathways similar to those seen in other tumours (Shim *et al*, 1998; Aft *et al*, 2002). Further studies will be needed to test this hypothesis.

In contrast to the situation with prostate cancer and other non-neural cancers (Tannenbaum, 1959; Bosland *et al*, 1999; Kritchevsky, 1999b; Mukherjee *et al*, 1999), little is known about the influence of diet on the progression of brain cancer. We found that orthotopic growth of the CT-2A brain tumour was similarly rapid during the unrestricted feeding of either a standard laboratory diet or a high-fat KD suggesting that a high-fat diet does not significantly enhance growth over that of a standard diet fed *ad libitum*. On the other hand, CT-2A growth was significantly reduced when either diet was restricted to 60% of the control diet. These findings indicate that orthotopic CT-2A brain tumour growth, like prostate tumour growth, is influenced more by the amount of dietary calories than by the origin or source of the calories (Tannenbaum, 1959; Mukherjee *et al*, 1999). Hence, diet and lifestyle may influence the progression of brain cancer.

An issue not addressed in this study is whether restricted feeding of either the SD or the KD would extend the survival of the CT-2A tumour-bearing mice. Survival studies are difficult with this rapidly growing brain tumour model because intracranial pressure, which often causes morbidity in brain tumour patients, is relieved due to skull expansion or tumour growth through the implantation burr hole (Mukherjee *et al*, 2002). This makes estimates of longevity capricious. Although the overall health was better in the R-fed mice than in the UR-fed mice, further studies will be necessary to determine if DR would extend survival.

In summary, our results suggest that experimental brain cancer is manageable through principles of metabolic control where plasma glucose levels are reduced and ketone body levels are elevated. Dietary energy restriction reduces tumour growth through effects on angiogenesis, apoptosis, and inflammation. Moreover, this dietary therapy may be effective for brain cancer management in humans and can be designed according to established standards (Nebeling and Lerner, 1995; Freeman *et al*, 2000). Body weight reductions of 10–12% should not adversely affect development or other functions in children as long as rate of weight gain is maintained (Nebeling and Lerner, 1995; Freeman *et al*, 2000; Greene *et al*, 2001). We also previously suggested that DR could reduce tumour-derived levels of procachexic factors by reducing tumour size (Mukherjee *et al*, 2002). Dietary caloric restriction may also be effective against recurrent human gliomas or could be used as an adjuvant with radiation or chemotherapy. We contend that dietary therapy may improve the clinical outcome of brain tumour patients and enhance their quality of life.
